# Peak hip and knee joint moments during a sit-to-stand movement are invariant to the change of seat height within the range of low to normal seat height

**DOI:** 10.1186/1475-925X-13-27

**Published:** 2014-03-12

**Authors:** Shinsuke Yoshioka, Akinori Nagano, Dean C Hay, Senshi Fukashiro

**Affiliations:** 1 Department of Life Sciences (Sports Sciences), The University of Tokyo, 3-8-1 Komaba, Meguro-ku, Tokyo, Japan; 2 Department of Computational Science, Kobe University, 1-1 Rokkodai-cho, Nada-ku, Kobe, Japan; 3 School of Physical and Health Education, Nipissing University, 100 College Drive, Box 5002, North Bay, Ontario, Canada

**Keywords:** STS, Low seat height, Minimum STS height test, Locomotive syndrome

## Abstract

**Background:**

Previous studies have consistently reported that decreasing seat height increases the peak hip and knee joint moments; however, these findings may not apply to biomechanical changes at very low seat heights. The purpose of this study, therefore, was to examine the effect of a large range of seat heights on peak joint moments of the lower limb during a sit-to-stand (STS) movement.

**Methods:**

Eight healthy young subjects participated in this experiment. Each subject was instructed to stand up from six seat heights (10, 20, 30, 40, 50 and 60 cm). Joint moments were calculated with an inverse dynamics method. The sum of the hip and knee joint moments was used as the index to indicate the mechanical load of the STS movement. The effect of seat height on the mechanical load was examined with both analytical and experimental approaches.

**Results:**

Through the analytical approach, it was revealed that the mechanical load of STS movements from low and normal seat heights (10 to 40 cm) always reaches its peak at or near the posture in which the thigh is horizontally positioned. This finding indicates that the peak value is invariant between the low and normal seat heights. Similar results were also found in the experimental approach. There were few significant differences in the peak mechanical load and the peak hip and knee joint moments between the low and normal seat heights, while they differed significantly between the low and high seat heights.

**Conclusions:**

This study concluded that, while the peak mechanical load and the peak hip and knee joint moments increase inversely to seat height within the range of high to normal seat height (60 to 40 cm), they are invariant to the change of seat height within the range of low to normal seat height (10 to 40 cm). These findings are useful for the design of chair, the improvement in the evaluation standard of minimum sit-to-stand height tests and the development of new muscular strength test.

## Introduction

A sit-to-stand (STS) movement, which is defined as a movement of standing up from a chair to an upright posture, is a frequently performed activity in daily living. Community-dwelling people stand up from a chair approximately 60 times each day [1]. Nonetheless, there are many elderly people who experience difficulty when standing up from a chair [2,3]. As such difficulties influence the quality of daily life and ability to remain independent, research on the STS task is important. Mechanical aspects of the STS task have been an area of particular focus, since it is one of the most demanding daily activities in mechanical terms [4-6].

Chair seat height is one of the most important determinants of a STS task [7], because it affects the peak hip and knee joint moments [6,8,9]. For example, showed that, when the seat height decreased from 64 cm to 43 cm, the peak hip and knee joint moment respectively increased 2.4 and 1.9 times [8]. showed that, when the seat height decreased from 115% subject’s knee height to 65%, the peak hip and knee joint moment of the right (left) leg respectively increased 1.1 (1.2) and 2.3 (1.7) times [6]. As shown in these previous studies, the consistent finding that decreasing seat height increases the peak hip and knee joint moments has been reported. focused on these mechanical characteristics and developed the minimum STS height test in which muscular strength is evaluated based on the minimum seat height from which a person can stand up [10]. In the test, a person who can stand up from a lower seat height is evaluated as a person who has higher muscular strength. Muranaga, 2001 [11] also developed a similar STS test [11]. The test is convenient in a general clinical site because it needs only four different height boxes (10, 20, 30 and 40 cm). Therefore, it is widely used as one item in the screening test for locomotive dysfunction known as locomotive syndrome in Japan [12].

However [13], recently reported that a small proportion (8-17%) of the variability in minimum STS height test was explained by knee extensor muscle strength [13]. Previous studies about the effect of seat height on joint moments [6,8,9] have focused on the seat height ranging from normal to high. In other words, no study has systematically examined the effect of seat height on joint moments over the range of low to high seat height. These lead to the hypothesis that, while the peak hip and knee joint moments increase inversely to seat height within the range of high to normal seat height, they are invariant to the change of seat height within the range of low to normal seat height. If the hypothesis is true, it may be necessary to revise the evaluation standards of the minimum STS height tests. Therefore, the purpose of this study was to examine the effect of seat height on the peak joint moments of lower limb over the range of low to high seat height with both analytical and experimental approaches.

## Methods

### Experimental data acquisition

Healthy young male (n = 6) and female (n = 2) subjects (mean (standard deviation): age 26 (3) years, body height 1.69 (0.10) m, body mass 63.1 (9.6) kg, knee height 0.46 (0.03) m) participated in this experiment with informed consent. None of them had any known musculoskeletal or neurological disorders. This project was performed under the approval of the ethics committee of the University of Tokyo.

Each subject was instructed to stand up from six seat heights and to repeat five trials per seat height except practice trials. Each subject performed two or three trials as practice. Seat heights were set at 10, 20, 30, 40, 50 and 60 cm. These heights are equivalent to 21.6 (1.4), 43.2 (2.8), 64.8 (4.2), 86.4 (5.6), 108.1 (7.0) and 129.7 (8.4)% of subjects’ knee height. Seat heights of 10, 20 and 30 cm were classified as low, the 40 cm height was normal, and seat heights of 50 and 60 cm were high. The experimental order of the six seat heights was randomized to each subject. Each subject was instructed to fold his/her arms on his/her chest and stand up from the chair without arm support. The movement strategy such as feet position, movement speed and movement pattern was not restricted. A brief rest time was assigned between trials. Throughout the current study, bilateral symmetry was assumed, and two-dimensional analyses on the sagittal plane were applied.

To obtain the kinematics, three-dimensional coordinates of the landmark points of the subject’s body were acquired using a 3D optical motion capture system with 7 cameras at 200 Hz (Hawk Digital System, Motion Analysis Corporation, Santa Rosa, CA, USA). Seven reflective markers were placed on the subject’s body (the right acromion, sacroiliac joint, right and left anterior superior iliac spines, right lateral epicondyle, right lateral malleolus and the distal end of the fifth metatarsal). All raw coordinate data were smoothed using a fourth-order Butterworth lowpass digital filter. The cutoff frequency (7 Hz) was determined with a residual analysis [14]. The hip joint center position was calculated from the sacroiliac joint, right and left anterior superior iliac spines and right lateral epicondyle [15]. The ground reaction force under the foot and the load imposed on the chair seat was measured with force platforms (9281B, Kistler Instrumente AG, Winterthur, Switzerland) placed under the foot and the chair, respectively. The force data were recorded at 1000 Hz.

The STS start and finish times were determined with the force platform. The time at which the vertical force fell outside three standard deviations of the vertical force during the static initial posture was regarded as the start time. The time at which the vertical force fell within three standard deviations of the vertical force during static upright standing posture was regarded as the finish time. The time at which the load imposed on the chair seat fell below 1 N was regarded as the seat-off time.

Hip, knee and ankle joint moments were calculated using an inverse dynamics method [14]. Joint moments of hip extension, knee extension and ankle plantar flexion were defined as positive. The joint moments were normalized by the mass of the whole body. The human body segmental parameters reported by de Leva (1996) [16] was used in the inverse dynamics calculation. The load imposed on a lower limb is small in the sitting phase of a STS movement, since the body is supported by the chair. Therefore, only the rising phase was analyzed.

Examined the minimum joint moment in order to achieve a STS task and revealed that the peak value of the sum of the hip and knee joint moments needed to be greater than 1.53 N.m/kg [17]. They also found that, while the individual joint moments were greatly affected by the movement patterns, the sum of the hip and knee joint moments was relatively invariant throughout the range of movement patterns. The sum of the hip and knee joint moments is an appropriate index to evaluate the “mechanical load” of a STS movement, since the effect of the movement pattern can be reduced. That is, the results can be evaluated purely from the view point of chair seat height. Therefore, [18] used the sum of the hip and knee joint moments as the index to indicate the mechanical load of a STS movement [18]. This index is also used in the current study.

The results of the current study are reported by using mean values and standard deviations. The peak joint moments were compared for the six seat height conditions by a one-way (seat height) analysis of variance (ANOVA) with repeated measures. The homoscedasticity of the data was checked and confirmed with Bartlett tests before an ANOVA. If the main effect was significant, pairwise comparisons were made for all pairs in the six seat height conditions by using Student’s paired t-test. To control the family-wise error rate in each multiple comparison, the *p*-value of each t-test was adjusted with Holm’s method [19], which is called a sequentially rejective Bonferroni procedure [20]. A statistical significance level was set at *p* < 0.05. All statistical analyses were processed with R language (version 3.0.1, R Foundation for Statistical Computing, Vienna, Austria).

### Analytical approach for the effect of seat height on mechanical load of a STS movement

The mechanical load, that is, the sum of the hip and knee joint moments about the transverse axis (Z axis) in the global coordinate system is derived with the following equations (Eq. 1-5) [21]. The variables indicating the trunk and shank inclinations are not involved in the equations. This means that the trunk and shank inclinations do not explicitly affect the sum of the hip and knee joint moments. This is why the sum of the hip and knee joint moments is invariant throughout the range of movement patterns (i.e. do not change with trunk and shank inclinations) and are therefore appropriate as an index to represent the mechanical load of a STS movement. On the other hand, the motion equations to derive individual hip and knee joint moments include variables related to the trunk and shank inclinations [17]. Therefore, the individual joint moments are affected by trunk and shank inclinations.

**Figure 1:**
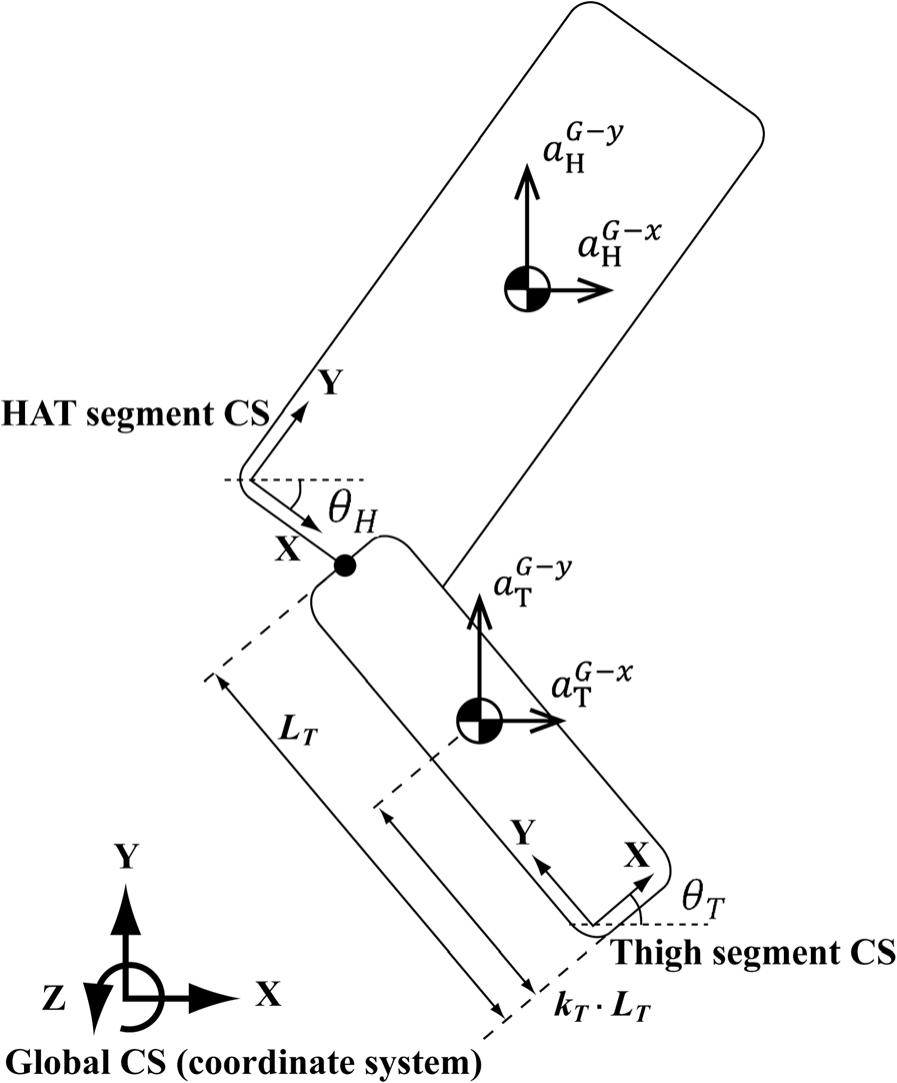
Definition of the coordinate systems and the parameters regarding head-arm-trunk (HAT) and thigh segments.

(1) Mechanical_Load=Term1+Term2+Term3+Term4

(2) $$\text{Term}1=\frac{m_{T}}{m_{B}}\cdot {\left(L_{T}\cdot r_{T}\right)}^{2}\cdot {\ddot{\theta }}_{T}$$

(3) $$\text{Term}2=\left(\frac{0.5\cdot m_{H}}{m_{B}}\cdot a_{H}^{G-x}+\frac{m_{T}}{m_{B}}\cdot k_{T}\cdot a_{T}^{G-x}\right)\cdot L_{T}\cdot \cos\theta_{T}$$

(4) $$\text{Term}3=\left(\frac{0.5\cdot m_{H}}{m_{B}}\cdot a_{H}^{G-y}+\frac{m_{T}}{m_{B}}\cdot k_{T}\cdot a_{T}^{G-y}\right)\cdot L_{T}\cdot \sin\theta_{T}$$

(5) $$\text{Term}4=\left(\frac{0.5\cdot m_{H}}{m_{B}}\cdot g+\frac{m_{T}}{m_{B}}\cdot k_{T}\cdot g\right)\cdot L_{T}\cdot \sin\theta_{T}$$

where x, y and z axes respectively represent forward, upward and right directions (Figure 1). *m*_*B*_, *m*_*H*_, *m*_*T*_ and *g* respectively represents the total mass of the body, the mass of HAT (head-arm-trunk) segment, the mass of thigh segment and the gravitational acceleration.  *k*_*T*_ represents the location parameter of the center of mass of thigh segment. *L*_*T*_ represents the length of the thigh segment. Coefficient of *m*_*H*_ is set to 0.5, because the HAT segment is supported by both legs during a STS movement and the load for each leg is one half of the value.  *θ*_*T*_ and ${\ddot{\theta }}_{T}$ respectively represent the angle and the angular acceleration of the thigh segment. *r*_*T*_ represents the parameter regarding the moment of inertia of the thigh segment about the center of mass, called the radius of gyration. $a_{\mathrm{Sg}}^{G-x}$ and $a_{\mathrm{Sg}}^{G-y}$ respectively represents the acceleration of the center of mass of segment Sg about the X and Y axis due to motion in the global coordinate system. The movements of each segment in the transverse and frontal planes were ignored, because a STS movement is bilaterally symmetrical and is primarily performed in the sagittal plane.

The contributions of Term1 and Term2 in Eq. 1 to the total (*Mechanical* _ *Load*) are usually negligible in a STS task, since the moment of inertia of the thigh segment is small and the anterior-posterior acceleration of the center of mass of the HAT and thigh segments are low compared to the vertical and gravitational ones. For example, in the case of STS movements obtained by [18], that of Term1, Term2, Term3 and Term4 was respectively 1, -2, 27 and 74 (%) (unpublished data). Therefore, Eq. 1 can be approximated by Eq. 6.

(6) $$\begin{array}{l} \text{Mechanical}\_\text{Load}\approx \text{Term}3+\text{Term}4\\ =\left\{\frac{0.5\cdot m_{H}}{m_{B}}\cdot \left(a_{H}^{\mathrm{Gy}}+g\right)+\frac{m_{T}}{m_{B}}\cdot k_{T}\cdot \left(a_{T}^{\mathrm{Gy}}+g\right)\right\}\cdot L_{T}\cdot \sin\theta_{T} \end{array}$$

Eq. 6 indicates that the mechanical load of a STS movement changes according to the two factors. One is the vertical accelerations of the HAT and thigh segments ($a_{H}^{\mathrm{Gy}}$ and $a_{T}^{\mathrm{Gy}}$), and the other is the sine of thigh inclination (sin *θ*_*T*_). The former reaches a peak soon after the seat-off [18], and the latter reaches maximum (sin *θ*_*T*_ = 1) at the posture in which the thigh is horizontally positioned (*θ*_*T*_ = *π*/2). These facts suggest that the maximum of the mechanical load is obtained under the condition that the thigh reaches horizontal position soon after the seat-off. It practically corresponds to the condition of the seat height ranging approximately from 20 to 30 cm. It is also suggested that the mechanical load changes subtly around 20 to 30 cm seat height, because the change of sin *θ*_*T*_ around *θ*_*T*_ = *π*/2 is subtle. For example, the change between *θ*_*T*_ = *π*/2 (approximately 20 to 30 cm seat height) and *θ*_*T*_ = 5*π*/12 (approximately 30 to 40 cm seat height) is only 3% (sin(*π*/2) = 1 and sin(5*π*/12) = 0.97). In the case of approximately 10 to 20 cm seat height (*θ*_*T*_ = 7*π*/12), the same is true (sin(7*π*/12) = 0.97). In summary, Eq. 6 indicates that the mechanical load of a STS task reaches its peak at or near the posture in which the thigh is horizontally positioned. The subjects assumed this posture when executing STS movements from low and normal seat heights. These indicate that the mechanical load of a STS movement is practically invariant to seat height changes between 10 and 40 cm. This result theoretically supports the hypothesis of the current study.

## Results

**Figure 2:**
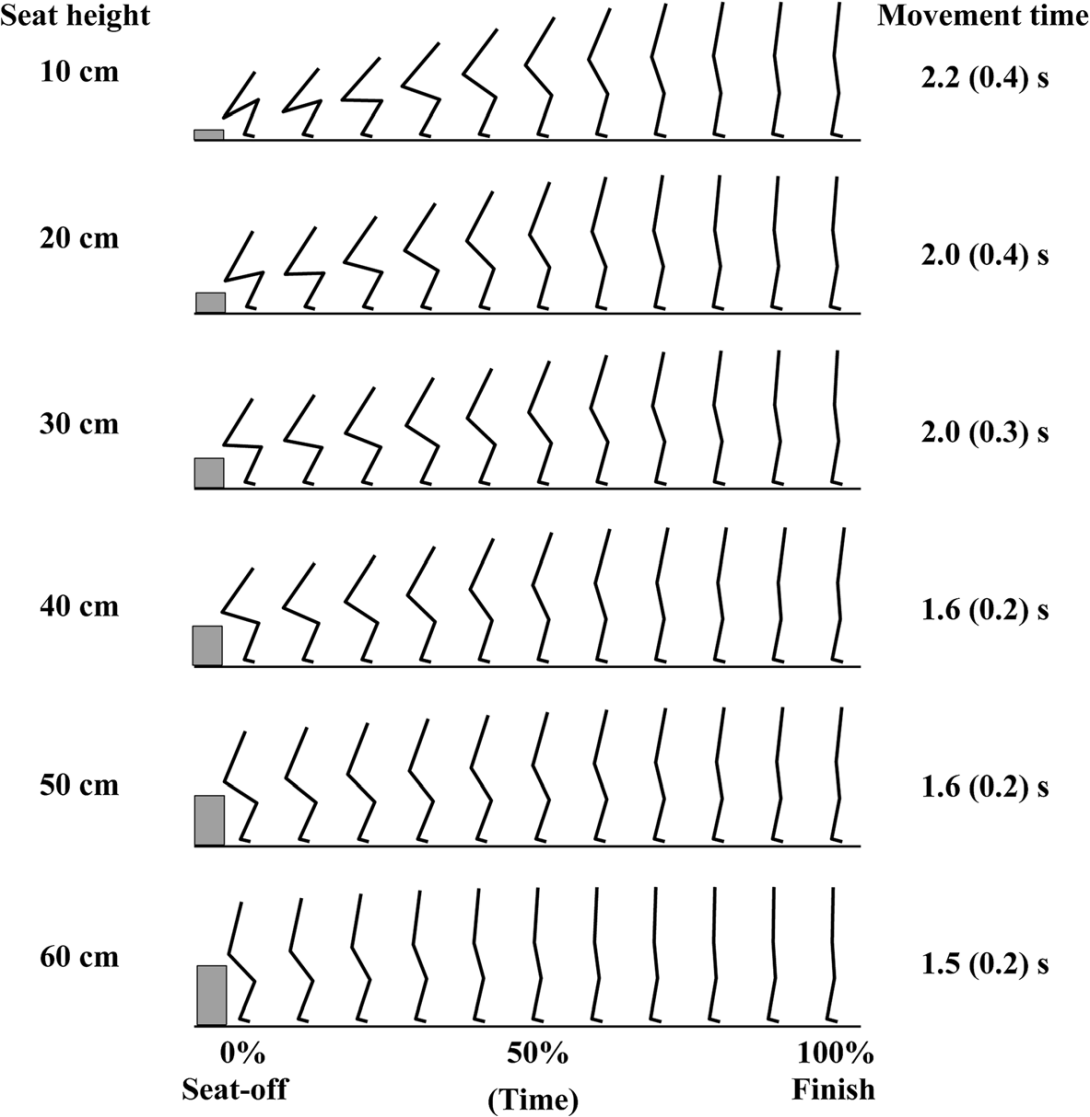
**Typical examples of STS movements from the seat heights of six different heights (10, 20, 30, 40, 50 and 60 cm).** Time = 0 (%) indicates the instance when the buttocks lost contact with the chair (seat-off time). Time = 100 (%) indicates the finish time of each STS movement. Stick figures were drawn at 10% time intervals. The gap between the center of hip joint and the surface of chair seat is due to the existence of the thickness between the center of hip joint and the skin surface of buttocks.

The movement times at the seat heights of 10, 20, 30, 40, 50 and 60 cm was respectively 2.2 (0.4), 2.0 (0.4), 2.0 (0.3), 1.6 (0.2), 1.6 (0.2) and 1.5 (0.2) seconds. All of the movement times were within the range of normal STS movement [22,23]. Figure 2 shows typical examples of STS movements from the seat heights of six different heights. The ankle dorsiflexion angle at the initial sitting position at the seat heights of 10, 20, 30, 40, 50 and 60 cm was respectively 13.4 (2.4), 19.3 (2.1), 20.1 (1.8), 17.3 (2.7), 17.2 (2.9) and 16.8 (3.3) degrees. These results were within the range of normal STS movements [24,25]. The maximum inclination angle of the HAT segment at the seat heights of 10, 20, 30, 40, 50 and 60 cm was respectively 39.1 (3.3), 38.6 (4.2), 39.1 (4.9), 34.6 (2.0), 28.6 (5.7) and 22.2 (5.7) degrees. Significant differences were found in the comparisons between the highest seat heights (50 and 60 cm) and all lower heights (10 to 40 cm). On the other hand, there was no significant difference among the seat heights of 10 to 40 cm.

**Figure 3:**
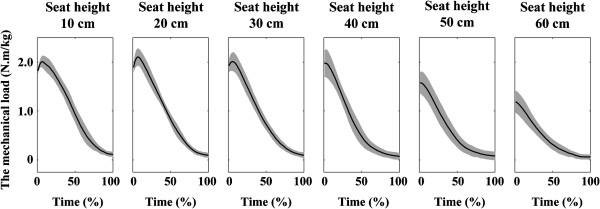
**Ensemble average of the mechanical load during STS movements from the seat heights of six different heights.** Time = 0 and 100 (%) respectively indicates the instance of seat-off and finish time. The grey area indicates ± 1 standard deviation of the ensemble average. The mechanical load reached a peak near the time of 0%.

**Figure 4:**
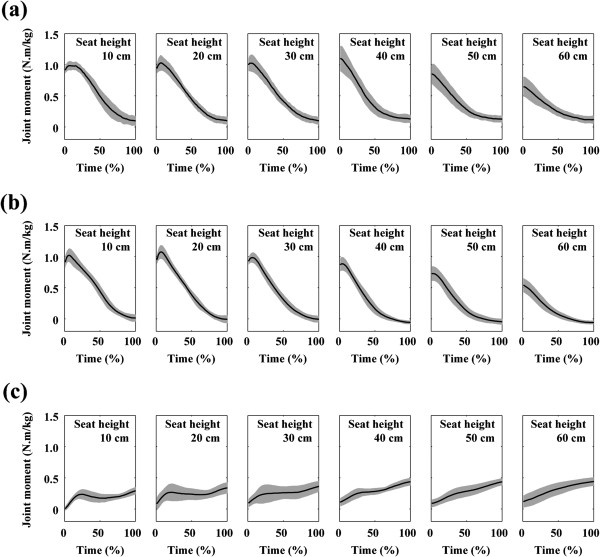
**Ensemble average of the joint moment during STS movements from the seat heights of six different heights: (a) hip, (b) knee and (c) ankle joint moments.** Time = 0 and 100 (%) respectively indicates the instance of seat-off and finish time. The grey area indicates ± 1 standard deviation of the ensemble average. The hip and knee joint moments reached a peak near the time of 0%. On the other hand, the ankle joint moment reached a peak near the time of 100%.

The mechanical load and the hip and knee joint moments reached a peak shortly after the seat-off in the cases of the seat heights of 10, 20 and 30 cm (Figures 3 and 4a and b) (the time of 0% indicates the instant of seat-off). In the cases of the seat heights of 40, 50 and 60 cm, they reached a peak at the instant of seat-off. On the other hand, the ankle joint moment reached a peak near the end of the movement (Figure 4c).

**Figure 5:**
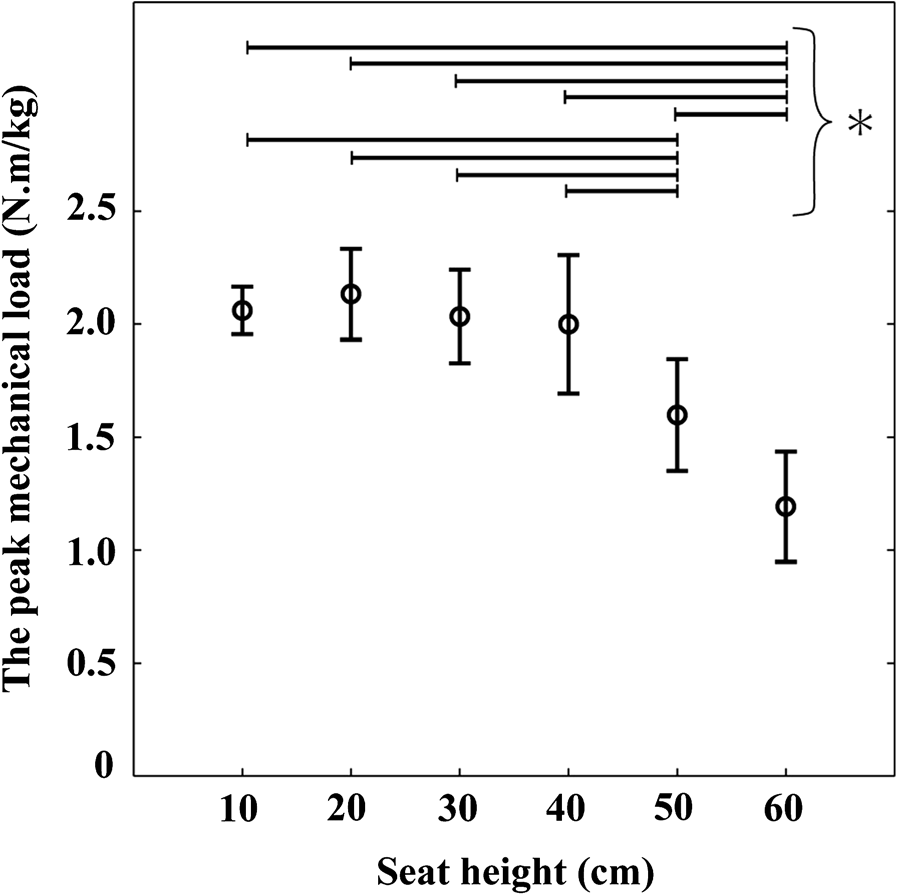
**Average and standard deviation of the peak mechanical load.** Asterisk mark (*) indicates significant difference (*p* < 0.05). The significant differences were found in the comparisons between the seat heights of 60 cm and lower than 60 cm (10 to 50 cm) and between the seat heights of 50 cm and lower than 50 cm.

**Figure 6:**
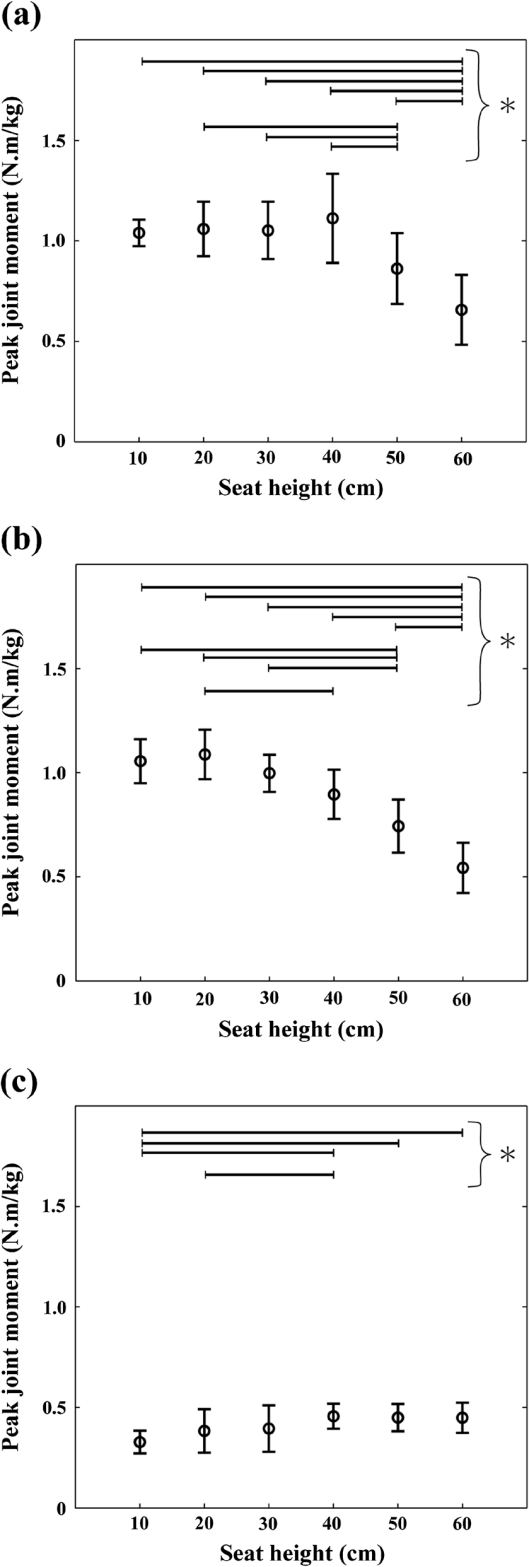
**Average and standard deviation of the peak joint moment: (a) hip, (b) knee and (c) ankle joint moments.** Asterisk mark (*) indicates significant difference (*p* < 0.05). In the cases of the hip and knee joints, the significant differences were mainly found in the comparisons between the seat heights of 60 cm and lower than 60 cm (10 to 50 cm) and between the seat heights of 50 cm and lower than 50 cm. On the other hand, in the case of the ankle joint, the significant differences were mainly found in the comparisons between the seat heights of 10 cm and greater than 40 cm.

The factor of seat height significantly affected the peak mechanical load and all of the peak joint moments: the mechanical load (main effect: F(5, 35) = 77.80, *p* < 0.001), the hip joint (main effect: F(5, 35) = 26.32, *p* < 0.001), the knee joint (main effect: F(5, 35) = 62.21, *p* < 0.001) and the ankle joint (main effect: F(5, 35) = 7.067, *p* < 0.001). In the statistical results of the mechanical load and the hip and knee joint moments, the sources of the significances were mainly found in the comparisons between the seat heights of 60 cm and lower than 60 cm (10, 20, 30, 40 and 50 cm) and between the seat heights of 50 cm and lower than 50 cm (10, 20, 30 and 40 cm) (Figures 5 and 6a, b). The significant differences were few among the seat heights of 10 to 40 cm. The peak hip and knee joint moments at the seat height of 40 cm were both 1.7 times as high as that at the seat height of 60 cm. In the case of the ankle joint moment, the sources of the significances were mainly found in the comparisons between the seat heights of 10 cm and greater than 40 cm (40, 50 and 60 cm). There was no significant difference for ankle joint moment among the seat heights of 30 to 60 cm.

## Discussion

We hypothesized that, while the hip and knee joint moments are inversely related to seat height within the range of high to normal seat height, they are invariant to the change of seat height within the range of low to normal seat height. To verify the hypothesis, we examined the effect of seat height on the peak joint moments of the lower limb over the range of low to high seat height. The finding about the invariance of the hip and knee joint moments within the range of low to normal seat height is unique to the current study.

### Validity of results

The hip and knee joint moments significantly increased as seat height decreased within the range of 60 to 40 cm (high to normal seat height) (Figure 6a and b). On the other hand, the ankle joint moment did not significantly change within the range (Figure 6c). These results are consistent with previous studies [6,8,9]. In the previous studies, the ratio of the hip (knee) joint moment at low seat height to that at high seat height ranged from 1.1 to 2.5 (1.7 to 2.0). The ratios of the current study were 1.7 in both the hip and knee joints and were within the range reported by the previous studies. These consistencies support the validity of the current study.

### Effect of seat height on hip and knee joint moments

The mechanical load, that is, the sum of the hip and knee joint moments significantly increased as seat height decreased within the range of 60 to 40 cm (Figure 5). On the other hand, it did not significantly change within the range of 10 to 40 cm (low to normal seat height). Taking the result obtained through the analytical approach (Eq. 6) into consideration, these statistical results indicate that, while the mechanical load of a STS task increases inversely to seat height within the range of high to normal seat height, it is practically invariant to the change of seat height within the range of low to normal. The individual hip and knee joint moments also had similar results. Yoshioka et al. (2007) has revealed that the individual hip and knee joint moments are affected by the inclination angle of the HAT segment [17]. In the current study, there was no significant difference for the angle among the seat heights of low to normal seat height (10 to 40 cm). This is why similar results were obtained even in the individual joint moments. These results verify the hypothesis of the current study.

It has been revealed that the inertia components of the sum of the hip and knee joint moments (Term1, Term2 and Term3 in Eq. 1) are negligible in the case of a slow STS movement (i.e. longer than 2.5 seconds) [18] such as the movement by the frail elderly [5,26] and the movement at a minimum STS height test. In those cases, Eq. 1 can be approximated by Eq. 7.

(7) $$\begin{array}{l} \text{Mechanical}\_\text{Load}\approx \text{Term}4\\ =\left(\frac{0.5\cdot m_{H}}{m_{B}}\cdot g+\frac{m_{T}}{m_{B}}\cdot k_{T}\cdot g\right)\cdot L_{T}\cdot \sin\theta_{T} \end{array}$$

Eq. 7 indicates that the mechanical load during a slow STS movement is a function of only the sine of thigh inclination (sin *θ*_*T*_). This means that the mechanical load of a slow STS task can be evaluated from the thigh segment angle at the instant of seat-off. If the angle is less than *π*/2 (the case of high seat height), the peak mechanical load occurring at the instant of seat-off is derived by substituting the angle into Eq. 7. If the angle is greater than *π*/2 (the case of low seat height), the peak mechanical load always occurs when the thigh is horizontally positioned. Therefore, the peak mechanical load is constant among chairs with low seat height and is derived from Eq. 8 consisting of only constant parameters.

(8) $$\left(\frac{0.5\cdot m_{H}}{m_{B}}\cdot g+\frac{m_{T}}{m_{B}}\cdot k_{T}\cdot g\right)\cdot L_{T}$$

When the body segmental parameters (*m*_*B*_ = 73.8 kg, *m*_*H*_ = 44.49 kg, *m*_*T*_ = 10.45 kg, *k*_*T*_ = 0.5905, *L*_*T*_ = 0.414 m and *g* = 9.80665 m/s^2^) and the thigh segment angle at the instant of seat-off (79 deg) in Yoshioka et al. (2007) [17] are substituted into Eq. 7, the value is 1.53 N.m/kg. This value corresponds to the minimum joint moment to achieve a STS task (1.53 N.m/kg) as reported. That is to say, Eq. 7 is the analytical solution to derive the minimum joint moment to achieve a STS task. Eq. 7 is applicable even with different subjects and seat heights, since the mechanics (the equation of motion) of a STS movement is common to different subjects and seat heights. These findings are useful for the mechanical explanation of a minimum STS height test. Also, they are useful as a convenient method of muscular strength evaluation for the frail elderly, since the muscular strength can be precisely evaluated only with an angle gauge.

### Effect of seat height on ankle joint moment

The peak ankle joint moment was also significantly affected by the change of seat height. However, the effect was limited mainly to the differences between the seat heights of 10 cm and greater than 30 cm (40 to 60 cm). Additionally, the magnitude of the change of the peak ankle joint moment (0.13 N.m/kg) was smaller than that of the peak hip and knee joint moments (0.45 N.m/kg and 0.54 N.m/kg). Therefore, these results indicated that the ankle joint is an invariant joint to the change of seat height.

Also, the following four results indicated that the main role of the ankle joint moment in a STS task is the maintenance of an upright posture rather than the lift of the body: 1) The ankle joint moment reached a peak near the end of a movement at which subject’s posture was almost upright (Figure 4c). 2) The peak ankle joint moment was correlated not to the vertical ground force at the instant that the ankle joint moment reached a peak (r = -0.12, not significant) but to the anterior-posterior position of the center of pressure at the same instant (r = 0.98, *p* < 0.001). 3) The magnitudes of the peak ankle joint moment (0.33 - 0.46 N.m/kg) (Figure 6c) are less than half the force exertion ability of the ankle joint at the plantar flexion angle of 0 deg even in the case of the elderly (1.07 N.m/kg) [27]. 4) Although the mechanical load increased with the decrease of seat height (Figure 5), the peak ankle joint moment inversely decreased (Figure 6c).

In summary, it can be said that the ankle joint is a joint invariant to the change of seat height, because the main role of the ankle joint moment in a STS task is the maintenance of an upright posture.

### Complementary characteristics of two kinds of sit-to-stand test

The result of the mechanical load indicates that minimum STS height tests are muscular strength tests suitable for the frail elderly who cannot stand up from the seat height of 40 cm, since the mechanical load is invariant within the range of the seat height of 10 to 40 cm. On the other hand, the STS tests based on movement time such as a 30-s chair-stand test [28] are muscular strength tests suitable for the people who can stand up from the seat height of approximately 40 cm and do not cover the frail elderly who cannot stand up from that seat height. These two tests have complementary characteristics with respect to the person to be measured. Therefore, if these two tests were to be combined, a wider range of people from the frail elderly to the young could be tested. This is an interesting future research theme.

## Conclusion

The findings of this study are as follows. (1) While the peak hip and knee joint moments increase as seat height decreases within the range of high to normal seat heights, they are invariant to the change of seat height within the range of low to normal. (2) The ankle joint is a joint invariant to the change of seat height because the main role of the ankle joint moment in a STS task is the maintenance of an upright posture. (3) The analytical solution of the mechanical load during a STS movement (Eq. 7) revealed that the mechanical load is a function of the sine of the thigh segment angle (vertical position: 0 degrees; horizontal position 90 degrees). (4) The minimum STS height tests and the STS tests based on movement time have complementary characteristics with respect to the person to be measured. These findings are useful for the design of chair, the improvement in the evaluation standard of minimum sit-to-stand height tests and the development of new muscular strength test.

## Competing interests

The authors declare that they have no competing interests.

## Authors’ contributions

SY performed the data collection and analyses, constructed the simulation model and drafted the manuscript. AN participated in the process of data collection, analysis, model construction and manuscript writing. DCH and SF contributed discussions and suggestions throughout this project, including the manuscript writing. All authors read and approved the final manuscript.
